# Will This Help Be Helpful? Giving Aid to Strangers in the United States and Japan

**DOI:** 10.3389/fpsyg.2021.784858

**Published:** 2022-01-25

**Authors:** Yu Niiya, Caitlin Handron, Hazel Rose Markus

**Affiliations:** ^1^Faculty of Global and Interdisciplinary Studies, Hosei University, Tokyo, Japan; ^2^Department of Psychology, Stanford University, Stanford, CA, United States

**Keywords:** helping, culture, uncertainty, models of agency, golden rule, platinum rule

## Abstract

Japanese rank among the least likely to intervene to help a stranger in a non-emergency situation while Americans rank among the most likely. Across four studies, we demonstrate that Japanese are less likely to offer help to strangers because their decisions rely more heavily on the assessment of the needs of others. Accordingly, when there is uncertainty about the need for help, Japanese are less likely to intervene than Americans because without an understanding of the needs of recipient, the impact of intervention may also be harmful. When the situation is unambiguous, Japanese and Americans are equally likely to help. This divergence in readiness to help strangers elaborates the understanding of why people in Japanese contexts are more likely than those in United States contexts to attend to the situation and to avoid uncertain situations. It also illuminates cultural differences in models of agency—implicit understandings of when and why a person should act to aid another.

## Introduction

Treat others the way **you** want to be treated—Golden Rule

Treat others the way **they** want to be treated—Platinum Rule

According to the Charities Aid Foundation World Giving Index (Charities Aid Foundation, 2018), the United States ranked among the top countries in helping behavior toward strangers, with 72% of respondents reporting that they had helped someone they did not know within the past month. In contrast, Japan, known for norms of interdependence and social attunement, ranked 142nd out of 144 countries. Why are people in Japanese cultural contexts less likely to help a stranger than people in American cultural contexts?

Here in four studies we address this question drawing on two widely known and multiply attributed moral “rules”—the Golden and the Platinum.^1^ Together these two normative prescriptions highlight a powerful distinction in the content and scope of an actor’s attention when considering whether to deliver aid to unknown others. In four studies, we use the helping-a-stranger paradigm to further probe the Japanese–American difference in conditioning one’s own actions on the perspective of others (e.g., Kim and Cohen, 2010; Riemer et al., 2014; Hashimoto, 2019), and to suggest that helping may not always have a prosocial connotation. “Helping” is an act of meaning (Bruner, 1990), and one that may well be perceived differently outside Western sociocultural contexts.

In choosing whether to deliver aid to another, the Golden rule urges potential helpers to imagine what they themselves would want or need in a situation. The Platinum rule, in contrast, urges potential helpers to do something different, that is, to take the perspective of the other—the third-person perspective rather than the first-person perspective—and to infer what the potential recipient may want or need in that particular situation. Deciding whether to act to aid another always requires considering the potential features of recipient and some aspects of the situation, but what exactly should be taken into account differs. Considered against one another, the Golden Rule and the Platinum Rule succinctly capture a difference in the often unmarked but undergirding models of agency that are predominant and that guide prosocial behavior in these two national contexts. This difference in behavioral orientation to others can further current understandings of American vs. Japanese differences in willingness to help strangers.

The many subtle and interrelated differences between people in North American and those in Japanese cultural contexts in both the content and the scope of their considerations when acting reflect divergent models of agency—implicit normative understandings of if, when and how to act (Kitayama et al., 2006; Markus et al., 2006; Stephens et al., 2009; Markus, 2016). In Japan (as in many contexts outside North America) the undergirding model of agency is one that assumes interdependence among people and that *being* is always experienced as *in relation to some others*. As a consequence, agency or acting in the world necessitates an ongoing awareness of others and adjusting to their needs and expectations of others (e.g., Rothbaum et al., 1982; Nisbett et al., 2001; Morling et al., 2002; Tsai et al., 2007; Gelfand et al., 2011). This model of agency leads actors to attend to and be particularly responsive to the inferred or known preferences of other people and the demands of the situation. In North American and many Western contexts, the predominant and undergirding model of agency is one that takes for granted the independence of free individuals and promotes the influencing of others and the environment rather than adjustment to them (Rothbaum et al., 1982; Morling et al., 2002; Tsai et al., 2007).

Specifically, we propose here that a decision to assist an unknown other invokes a somewhat different set of considerations in Japan than in the United States. Since Japanese cultural contexts like many East Asian contexts tend to foster the understanding that the source and the consequences of actions are always dependent on the specific situational context (Choi and Nisbett, 1998; Markus et al., 2006; Markus and Conner, 2014), deciding to intervene to help a stranger in a public situation can be particularly complex. People need to assess the situation and to focus on whether their actions are needed, wanted, and actually helpful. In other words, when a situation is ambiguous in Japan, people will be particularly likely to invoke a version of the Platinum Rule, and to ask, how would *this person want to be treated in this situation*, and “will my help be helpful?”

The United States American cultural context, by contrast, tends to promote the understanding that peoples’ actions are and should be independent of particular situations, and that actions should be driven primarily by the actor’s personal preferences, motivations, and capacities (e.g., Choi and Nisbett, 1998; Miller et al., 2011, 2014; Riemer et al., 2014; Eom et al., 2016). In determining whether to aid another, people are more likely than in Japanese contexts to invoke some version of the Golden Rule, and to ask how would *I want to be treated*, and *do I have the capacity or desire to help*, and less on what might be the consequences for the recipient.

Among the most robust findings in cultural psychology are those that demonstrate the relatively greater concern among people in many East Asian contexts compared with those in European American contexts with the particulars of the situation when they explain the sources of behavior or predict future behavior (e.g., Kim and Cohen, 2010; Riemer et al., 2014; Hashimoto, 2019). A further well-validated distinction between East Asian and North American cultural contexts is a relatively greater concern with the face of the other, i.e.,—a concern with the other’s status and feelings—as opposed to dignity—a concern with self-worth (e.g., Oetzel and Ting-Toomey, 2003; Kim and Cohen, 2010; Smith et al., 2021). In many East Asian cultural contexts, especially public ones, it is essential to take others’ perspective on one’s potential actions; one’s own assessments are insufficient.

Among the many factors that comprise “the situation,” chief among them in a decision of whether to aid another is a refraction of the situation through the eyes of the potential recipient, and some consideration of what that recipient might need or want in that situation. By contrast, in dignity cultures, the worth or goodness of one’s actions are not supposed to be defined by others’ views and evaluations but instead by one’s own (Kitayama et al., 2004; Hoshino-Browne et al., 2005; Kim et al., 2010; Leung and Cohen, 2011). The majority of research relevant to cultural variation in providing aid and support to others has focused on support seeking or support giving among close others or people known to each other and has found that the meanings and functions of social support differ across cultures (Chen et al., 2012; Mojaverian and Kim, 2013). Here we extend this research to consider the situation of unsolicited aid provided to strangers.

In Japan, where the view of others and the situation are often central in determining who should take what action and when, these expectations are rarely explicit. People often hesitate to verbalize their desires or needs out of consideration of others (*enryo*; Ishii, 1984; see also Kim et al., 2008). People are expected to guess, sympathize with, and understand others’ situation without being told what their needs are (*sasshi*; Hamaguchi, 1977). These *enryo-sasshi* interactions work when people share sufficient context (Miike, 2003) or when social norms for a given situation are very clear. With strangers, however, people have little shared contexts to guess their need, rendering it difficult for Japanese to draw on *sasshi* to determine the right action to take.

Taking action under uncertainty is also riskier in Japan. Transgressions and their impact are weighted more heavily than the intention behind an action (Feinberg et al., 2018). Further, receiving help can create a debt for the recipient, and Japanese worry about repaying these social debts more than do Americans (Miller et al., 2017). Giving inadequate or unwanted help could harm, embarrass, or indebt others (Miller et al., 2017). Thus, in Japan, intervening in a situation to provide aid to a stranger may not be as closely associated with the positive, prosocial concept of “helping” as it is for Americans. As unneeded, unwanted, or poorly executed help may do more harm than good, in Japan what is most “helpful” may be to do nothing (Mitsuno and Miura, 2010; Yamamura, 2013).

### Research Overview

In four cross-cultural studies, we drew on the framework of models of agency to predict and explain how Japanese offer aid to strangers less readily than Americans. We hypothesize that people in Japanese contexts are less likely than people in American contexts to offer help especially when the situation of the recipient is ambiguous because in Japan a decision to intervene depends relatively more heavily on the demands of the situation and others’ need for help. Specifically, in Study 1, we asked people in Japan and the United States to provide open-ended explanations for why they would not intervene to help a stranger in a given situation and tested whether Japanese compared to Americans would be more likely to refer to the wants and needs of other people (Hypothesis 1). In Study 2, we varied the ambiguity surrounding the needs for help and tested whether people in Japanese contexts were less likely than people in American contexts to offer help to a stranger when the situation was ambiguous (Hypothesis 2) and whether this cultural difference diminished as the need for help became more certain (Hypothesis 3). Study 3 tested Hypotheses 2 and 3 using a between-subject design. It also tested whether Japanese perceived greater ambiguity in a given situation than Americans and whether perceived ambiguity accounted for the cultural difference in helping (Hypothesis 4). Finally, in Study 4, participants in Japan and the United States imagined taking actions to help strangers that resulted in either positive, negative, or neutral outcomes and rated how much the action was helpful and successful, and also how responsible they felt for the impact of the action. Considering that in Japan, a person’s action takes meaning primarily within the situation with its many norms and expectations, we predicted that when a stranger’s needs and wants are not explicit, Japanese will perceive their intervention to be less helpful and successful than Americans, even when the outcome is positive (Hypothesis 5).

## Study 1: Why Didn’t You Help?

Study 1 asked Japanese and American participants to provide open-ended explanations for why they would not help a stranger in a given situation. We tested whether in Japan, people were more likely than in the United States to spontaneously refer to the target’s wants and needs (Hypothesis 1).

### Method

#### Participants

In Study 1, we targeted 200 participants (100 from each culture) because we figured this would yield the maximum number of responses that two bilingual coders could cover (200 participants providing 4 reasons each results in 800 responses). For the United States sample, 113 participants were recruited through Amazon’s MTurk to take part in a survey on everyday experiences. Data from 27 participants were excluded from the analysis either because they wished their data to be withdrawn (*n* = 5), did not provide the free responses (*n* = 2), or provided incomprehensible free responses (*n* = 20). The final sample consisted of 86 respondents (44.0% female), with a mean age of 37.87 (*SD* = 12.19). Three participants indicated they were not born in the United States, but all have lived in the United States more than 22 years. The majority reported being White or European American (79.1%), followed by Asian/Asian American (9.3%) and Black/African American (8.1%).

For the Japanese sample, 102 participants were recruited through Lancers, a Japanese crowdsourcing company. Data from six participants were excluded from the analysis either because they wished to have their data withdrawn (*n* = 5) or did not provide the free response (*n* = 1). The final sample consisted of 96 respondents (39.6% female) with a mean age of 42.32 (*SD* = 10.99). All of them indicated their nationality as Japanese.

#### Materials

Participants read the following scenario: “Imagine you are walking to meet a friend. You are early for the appointment. As you’re walking, a person ahead of you trips over a curb and falls to the ground. You are unsure whether the person is injured but you pass by without stopping.” Participants then provided an open-ended response to the following questions: “What are the first two reasons that come to mind for why you would not stop?” and “What are the next two reasons that come to mind for why you would not stop?” Participants were given separate answer boxes for each of the reason. Participants completed a measure of uncertainty avoidance (Jung and Kellaris, 2004; e.g., “I prefer structured situations to unstructured situations;” α_United States_ = 0.85, α_Japan_ = 0.81) and demographic information.

#### Coding

The American responses were coded by the second author whose native language is English, the first author who is proficient in both languages, and another bilingual coder who was blind to the hypotheses. The Japanese responses were coded by the two bilingual coders. We ensured consistent coding across the two samples by having the two bilingual coders code all responses. One bilingual coder was blind to the purpose and the hypotheses of the study. We had three categories derived from our theory (i.e., the needs of the potential recipient of help, the recipient being a stranger, and burden or risk associated with helping) but after reading a sample of responses, the coders agreed to add four thematically derived categories (i.e., late for appointment, social situation, attributes of the recipient, and attributes of the helper; see Table 1). Then, the coders independently coded 30 responses and discussed the discrepancies to ensure that they shared the same coding criteria. We compared the coding and computed the reliabilities among the three coders in the United States (κ_us_ = 0.84) and among two coders in Japan (κ_Japan_ = 0.92). The coders discussed and solved the inconsistencies.

**TABLE 1:** Why didn’t you help? Japanese participants were more likely to describe their non-intervention in terms of the needs of the stranger and American participants in terms of personal attributes of helper and of the stranger (Study 1).

		First reason for non-intervention				Overall reasons for non-intervention			
Category	Examples	United States% (*n* = 86)	Japan% (*n* = 96)	*Chi-square*	*p*	United States (*n* = 343)	Japan (*n* = 381)	*Chi-square*	*p*
Needs of the potential recipient	It could have been an unwelcome favor. The person didn’t really have a serious injury and it wasn’t that bad.	18.6	36.5	7.169	0.007**	17.8	26.2	7.141	0.008**
Late for appointment	I am eager to just meet up with my friend already.	23.3	27.1	0.352	0.553	15.7	20.7	2.827	0.093
Stranger	It would feel awkward to talk to a stranger.	4.7	11.5	2.779	0.095	4.7	8.1	3.497	0.061
Burden or risk	I didn’t want to be held up if they were injured and needed my help.	2.3	9.8	3.970	0.046*	9.9	10.2	0.016	0.899
Social situation	I wouldn’t get involved because I’m sure someone else will help. Someone else already stopped to help them.	8.1	7.3	0.046	0.830	7.6	14.4	7.919	0.005**
Attributes of the potential recipient	They looked dirty. The stranger could be dangerous and armed.	20.9	6.3	8.540	0.003**	17.2	10.0	7.774	0.005**
Attributes of the helper	I am lazy. I am having a bad day, and I am feeling selfish. I’m not strong enough to help them up.	18.6	4.2	9.667	0.002**	22.7	8.4	26.586	<0.001**
Other (unclassifiable)	I would have stopped!	3.5	0.0			4.4	1.8		

*p < 0.05, **p < 0.01.

### Results and Discussion

Because we were interested in the percentage of participants from each cultural group who would spontaneously report the ambiguity of other’s need as a reason for non-intervention, we examined the first reason participants brought up (86 in the United States and 96 in Japan). As shown in Table 1, the needs of the potential recipient was the most frequent reason for not helping in Japan (36.5%), followed by being late for the appointment (27.1%), the target being a stranger (11.5%), and the burden or risk associated with helping (9.8%). For Americans, the most frequent first reason was being late for appointment (23.3%), followed by the attributes of the potential recipient (20.9%), the attributes of the helper (18.6%), and the needs of the potential recipient (18.6%). Japanese were more likely than Americans to claim that they did not help because their help was not needed or wanted (36.5% vs. 18.6%; χ^2^ = 7.17, *p* = 0.007). Consistent with Hypothesis 1, more Japanese than American respondents reported basing their decision on what others wanted or needed in this situation. In contrast, American respondents were more likely than Japanese to mention attributes of the potential recipient (20.9% vs. 6.3%; χ^2^ = 8.54, *p* = 0.003) or the helper (18.6% vs. 4.2%; χ^2^ = 9.67, *p* = 0.002) as their reason for not helping. Thus, for Americans, the decision to intervene or not focused on whether they had the intention or the capacity to help or on the deservingness of the recipient. Americans and Japanese referred to being late for the appointment to a similar extent (23.3% and 27.1%; χ^2^ = 0.35, n.s.), presumably because of the salience of the situational demand (Darley and Batson, 1973).

Though Japanese were somewhat more likely than Americans to refer to the burden or risks associated with helping (9.8% vs. 2.3%; χ^2^ = 3.97, *p* = 0.046) or the target being a stranger (11.5% vs. 4.7%; χ^2^ = 2.78, *p* = 0.095), these numbers were overall relatively low. These findings suggest that Japanese decisions to not intervene were not merely a result of indifference toward a stranger’s suffering or avoidance of a stranger but in many cases rooted in an assessment of whether their actions would be useful. Notably and consistent with this finding, a general measure of uncertainty avoidance did not differ between the United States (*M* = 3.11, *SD* = 0.88) and Japan (*M* = 3.13, *SD* = 0.71), *t*(180) = −0.11, *p* = 0.92.

The pattern of the results was similar when we analyzed the overall reasons participants provided (see the right columns in Table 1). Japanese responses were more likely than American responses to refer to the needs of the potential recipient (26.2% vs. 17.8%, χ^2^ = 7.14, *p* = 0.008), whereas American responses were more likely than Japanese responses to refer to attributes of the potential recipient (17.2% vs. 10.0%; χ^2^ = 7.77, *p* = 0.005) or the helper (22.7% vs. 8.4%; χ^2^ = 26.59, *p* < 0.001). No difference was found for being late for appointment (15.7% in the United States vs. 20.7% in Japan; χ^2^ = 2.83, *p* = 0.093), the recipient being a stranger (4.7% in the United States vs. 8.1% in Japan; χ^2^ = 3.50, *p* = 0.061), and the burden or risks (9.9% in the United States vs. 10.2% in Japan; χ^2^ = 0.02, *p* = 0.899). The cultural differences remained significant even when encouraged to provide multiple reasons for the non-intervention. Although the percentages of participants mentioning social situation as their first reason for non-intervention were similarly low in both cultures (8.1% in the United States vs. 7.3% in Japan; χ^2^ = 0.05, *p* = 0.830), in the overall analyses, social situations were more frequently mentioned in the Japanese than American responses (7.6% in the United States vs. 14.4% in Japan; χ^2^ = 7.92, *p* = 0.005), reflecting perhaps Japanese greater attunement to social situations.

## Study 2: Are Japanese More Attuned to Others’ Needs for Help?

Study 2 was designed as specific tests of whether a Japanese decision to offer help to a stranger depended more on their assessments of others’ needs for help than Americans. We varied the ambiguity surrounding the needs for help and tested whether cultural differences in helping are more salient in more ambiguous situations. Specifically, we predicted that people in Japanese contexts are less likely than people in American contexts to offer help to a stranger in ambiguous situations (Hypothesis 2) but that this cultural difference would diminish as the need for help becomes more certain (Hypothesis 3).

### Method

#### Participants

Participants in the United States and Japan completed a survey on interpersonal goals that was designed for different studies. The number of participants was thus determined based on the need of those different studies. In the United States, 709 participants were recruited through Amazon’s MTurk to take part in a survey on interpersonal goals. The questions about helping were included at the end of the survey. Data from 111 were excluded because participants either failed the attention check question (*n* = 22) or had zero variance on at least one of the five pages that measured their interpersonal goals (*n* = 89). The final sample consisted of 598 participants (41.8% women), with age ranging from 19 to 78, a mean of 35.60 (*SD* = 11.39), and a median of 33. Participants identified their ethnicity as European or White (75.9%), African American or Black (11.7%), Latino(a) or Hispanic (7.4%), or Asian (6.0%). Most of them (93.8%) indicated having lived in the United States their entire lifetime, while 36 indicated having lived in the United States between 2 and 60 years, with a mean of 26.31 years (*SD* = 14.81) and a median of 24. Of these, seven reported having lived in Japan, with a range between 0 and 5 years.

For the Japanese sample, 2413 participants were recruited using Ann to Kate service provided by a Japanese web survey company, Marketing Applications, Inc. Participants completed questions about interpersonal goals and helping as part of a pre-screening survey for a different study. From this, we excluded 568 participants who failed the attention check question and 255 who had a zero variance on at least one of the five pages that measured their interpersonal goals. The final sample consisted of 1590 participants (54.8% women), ranging in age from 20 to 81, with a mean of 41.87 (*SD* = 14.51) and a median of 41.

#### Materials

Participants read two scenarios with ten iterations each. In the pregnant scenario, they read: “Imagine you are sitting in a crowded train. A woman who “looks pregnant” is standing in front of you.” Participants were then asked to rate how likely they would be to give up their seat on scales from 1 (*I will definitely not offer seat*) to 5 (*I will definitely offer seat*) in 10 iterations that varied from the least ambiguous (“when the likelihood of the woman being pregnant is 100%”) to the most ambiguous (“when the likelihood of the woman being pregnant is 10%”) in decrements of 10%. In the sickness scenario, participants rated how likely they would offer help to a person squatting on the side of a road on scales from 1 (*I will definitely not offer help*) to 5 (*I will definitely offer help*) in ten iterations that varied from most ambiguous (“when the likelihood of this person being sick was 10%”) to least ambiguous (“when the likelihood of this person being sick was 100%”) in increments of 10%. The questionnaire was first developed in Japanese, then translated into English by the first author and back translated by a bilingual psychologist.

### Results and Discussion

**FIGURE 1:**
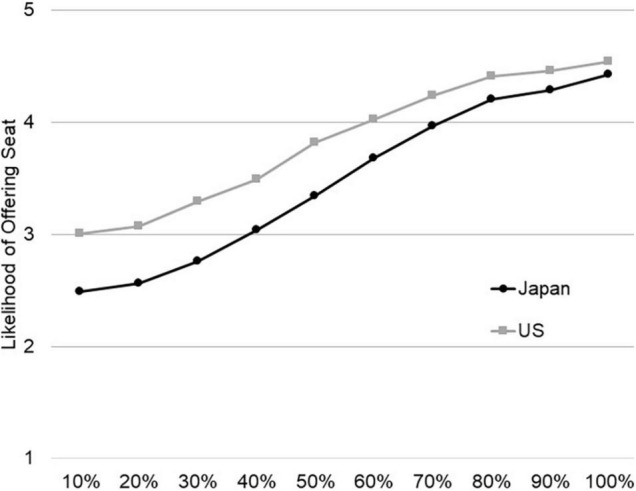
Japanese are less likely than Americans to offer seat to a woman when the need for help is ambiguous, but the difference diminishes as the likelihood of the woman being pregnant increases from 10% certain to 100% certain (Study 2).

A multilevel modeling was conducted separately for each scenario to see whether the ambiguity of the stranger’s need was associated with the likelihood of offering help and whether this association differed by culture. As shown in Figure 1, culture (0 = United States, 1 = Japan) had a main effect, such that Japanese were less likely to offer a seat to the woman than Americans, *b* = −0.58, *t*(2147.78) = −10.47, *p* < 0.001, 95% CI [−0.68, −0.47]. Moreover, as expected, people were more likely to offer their seat as the clarity of the need increased, *b* = 0.19, *t*(2121.31) = 30.87, *p* < 0.001, 95% CI [0.18, 0.21]. More importantly, the slope varied by individuals, Wald *Z* = 30.18, *p* < 0.001, *b* = 0.022, 95% CI [0.022, 0.023]. The Culture × Ambiguity interaction suggested that the association between ambiguity about the need for help and the likelihood of offering help was stronger among Japanese than among Americans, *b* = 0.05, *t*(2121.99) = 6.81, *p* < 0.001, 95% CI [0.04, 0.06]. Consistent with Hypotheses 2 and 3, cultural differences, which were the most pronounced at the highest level of ambiguity (*M*_United States_ = 3.01, *SD*_United States_ = 1.35; *M*_Japan_ = 2.49, *SD*_Japan_ = 0.95, *t*(2168) = 10.07, *p* < 0.001, *g* = 0.48, 95% CI [0.39, 0.58]), diminished as the ambiguity of the needs decreased, until it almost disappeared when the need was 100% clear (*M*_United States_ = 4.54, *SD*_United States_ = 0.86; *M*_Japan_ = 4.43, *SD*_Japan_ = 0.80, *t*(2175) = 2.91, *p* = 0.004, *g* = 0.13, 95% CI [0.04, 0.23]).

**FIGURE 2:**
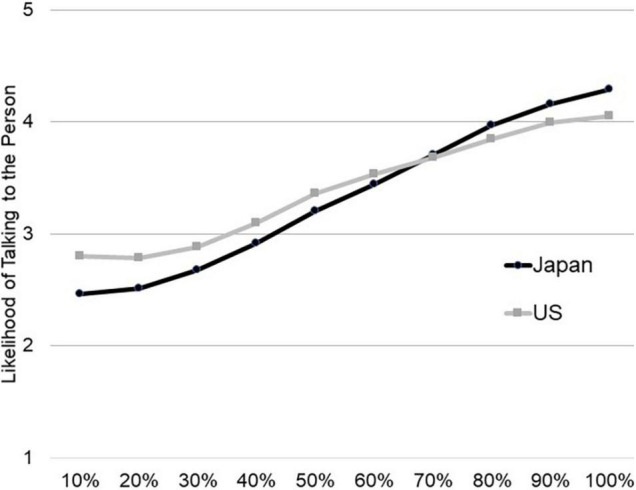
Japanese are less likely than Americans to assist a person sitting on the street in an ambiguous situation, but the difference diminishes and reverses as the likelihood of the person being sick increases from 10% certain to 100% certain (Study 2).

A similar result emerged for the second scenario. As shown in Figure 2, Japanese respondents were less likely than Americans to offer help to a potentially sick person, *b* = −0.34, *t*(2150.27) = −5.98, *p* < 0.001, 95% CI [−0.45, −0.23] and reducing the ambiguity of the needs predicted greater likelihood of offering help, *b* = 0.16, *t*(2133.21) = 24.22, *p* < 0.001, 95% CI [0.15, 0.18]. The slopes differed between individuals (Wald *Z* = 30.62, *p* < 0.001, *b* = 0.026, 95% CI [0.024, 0.027]), such that the slopes were more pronounced for Japanese than for Americans, *b* = 0.06, *t*(2133.52) = 7.84, *p* < 0.001, 95% CI [0.05, 0.08]. Although Japanese (*M* = 2.46, *SD* = 1.02) were less likely than Americans (*M* = 2.77, *SD* = 1.25) to offer help in the most ambiguous situation, *t*(2179) = 5.88, *p* < 0.001, g = 0.29, 95% CI [0.19, 0.38], this cultural difference disappeared when the situation was 70% certain (*M*_United States_ = 3.68, *SD*_United States_ = 1.30; *M*_*JP*_ = 3.71, *SD*_*JP*_ = 1.07, *t*(2173) = −0.51, *p* = 0.61), and even reversed, such that Japanese (*M* = 4.29, *SD* = 0.99) were more likely to offer help than Americans (*M* = 4.05, *SD* = 1.23) in the most certain situation, *t*(2170) = −4.75, *p* < 0.001, *g* = −0.23, 95% CI [−0.32, −0.13]. These results support the idea that the ambiguity about others’ needs for help has a greater impact on the likelihood to help a stranger among Japanese than Americans and that Japanese sensitivity to the needs of others explains why Japanese are less likely to help a stranger.

## Study 3: Is This a Helping Situation?

**FIGURE 3:**
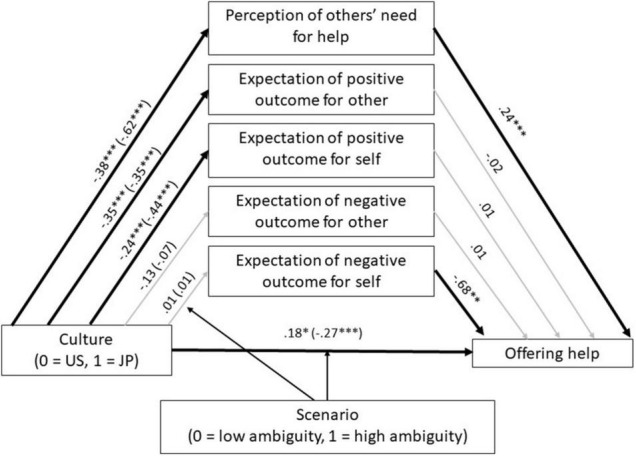
Perceived need for help and expectations of outcomes for others and self accounting for the cultural difference in offering help to a stranger in low and high ambiguity situations (Study 3). Values are unstandardized coefficients. Values in parentheses are for high ambiguity scenarios. Arrows in bold indicate paths that were significantly moderated by scenario. ^∗^*p* < 0.05,^∗∗^*p* < 0.01, ^∗∗∗^*p* < 0.001.

Study 3 aimed to conceptually replicate the findings of Study 2 that cultural difference in helping emerges especially when the need of help is ambiguous (Hypotheses 2 and 3). More importantly, it examined whether the perceived needs for help explained the cultural difference in helping, especially in ambiguous situations (Hypothesis 4). Study 2 manipulated ambiguity about the needs for help by varying the likelihood that the target was pregnant or sick but in reality, people also differ in how much they perceive others’ needs in a given situation. For some, a person with a 60% likelihood of being sick may be perceived as definitely needing help but for others, as still showing ambiguous needs. Based on the results from Study 2, we expected that, in a given situation, Japanese perceive greater ambiguity in the needs of others than Americans, such that Japanese would be less certain the target wants or needs help, and also less certain that their intervention would be beneficial to the target. We expected a moderated mediation such that these cultural differences would be particularly salient in high rather than low ambiguity situations (see Figure 3). When the situations are ambiguous, we predicted that perceived ambiguity would mediate the effect of culture on helping a stranger. When the situation was clear, we did not expect much cultural difference in people’s tendency to offer help and that there would be no indirect effect through perceived ambiguity. We also aimed to rule out the possibility that Japanese lower tendency to intervene was rooted in their motivation to avoid negative consequences for the self. In addition, we changed the within-subject design of Study 2 to a between-group design to reduce the possibility of demand characteristics and increased the number of items to measure the likelihood of offering help.

### Method

#### Participants

Our target sample size was 400 in each cultural group. Following Fritz and MacKinnon (2007), it was determined that we would need around 300 participants from each culture to detect an indirect effect in which the effect sizes of the first path (from the predictor to the mediator) and the second path (from the mediator to the outcome variable) are expected to be small (0.14). Because we wanted to test whether these indirect effects were moderated by the ambiguity of the situation, we increased each sample by 100. In the United States, 402 participants were recruited through Amazon’s MTurk. Five participants wanted their data withdrawn at the end of the survey, resulting in a final sample of 397 (43.6% women). Their age ranged from 18 to 74 with a mean of 40.7 (*SD* = 10.28) and a median of 30. The majority (79.6%) indicated their ethnicity as White or European American, 9.8% as Hispanic or Latino(a), 6.3% as Black or African American, and 3.0% as Asian or Asian American. All of them indicated they have lived in the United States their entire lifetime, except seven participants who indicated they have lived in the United States between 17 and 63 years. In Japan, 402 participants were recruited through Lancers, of which three indicated the desire to withdraw their data, resulting in a final sample of 399 (51.9% women). Their age ranged from 18 to 75 with a mean of 40.71 (*SD* = 10.28) and a median of 41. All of them indicated their nationality as being Japanese, except one who indicated otherwise (Korean).

#### Scenarios

Participants read one of the following three scenarios. In the low ambiguity scenario, the target explicitly asked for assistance (“*Imagine you are walking in your neighborhood. Ahead of you, you see a tourist who seems lost. The tourist comes to you and asks for directions*”). In the two high ambiguity scenarios, there was no explicit mention of the target seeking help (“*Imagine you are walking down a semi-crowded street. Ahead of you, a person trips over a curb and falls to the ground. You are not sure if the person is hurt or not*” and “*Imagine you are on an airplane. Ahead of you is a person who is struggling to put their luggage in the overhead bin*”). The low ambiguity tourist scenario was coded as 0 and the two high ambiguity scenarios (tripping and luggage) were coded as 1.

#### Measures

Participants rated on scales from 1 to 5 how likely they would offer help to the stranger with three items (α_United States_ = 0.75, α_Japan_ = 0.76): “How likely would you be to help the person *up from the ground* in this situation?” “How hesitant would you be to help the person *up* in this situation? (*reversed*)” and “How stressful would it be to make the decision to help or not in this situation? (*reversed*)” They also rated the perception of other’s need for help with two items (α_United States_ = 0.72, α_Japan_ = 0.79): “How much do you think the person needs help *up* in this situation?” and “If you were the person *who tripped*, how much would you want to be helped?” Positive outcome of one’s action for the target was measured with a composite of one item (“How likely is it that there would be positive outcomes to the person if you were to help in this situation?”) and ratings of how positively the target would feel (e.g., “grateful;” α_United States_ = 0.81, α_Japan_ = 0.82). Positive outcome for the self was measured with a composite of one item (“How likely is it that there would be positive outcomes to you if you were to help in this situation?”), ratings of how positively they would feel (e.g., “appreciated”) and how positively their help would be perceived (e.g., “supportive;” α_United States_ = 0.86, α_Japan_ = 0.85). Similar scales were used to measure negative outcomes for the self (α_United States_ = 0.95, α_Japan_ = 0.91) and other (α_United States_ = 0.86, α_Japan_ = 0.87). Participants used scales ranging from 1 (*extremely unlikely*) to 5 (*extremely likely*). See Supplementary Material for the full list of items.

The questionnaire was first developed in English, then translated into Japanese by a bilingual psychologist, and back translated by another bilingual psychologist. The two bilingual psychologists and one native speaker of English discussed to solve any discrepancy in the translations. The questionnaires also contained other questions not related to this study.

### Results and Discussion

First, using PROCESS model 1 (Hayes, 2018), we tested whether Japanese respondents were less likely than Americans to help a stranger when situational ambiguity was high rather than low (0 = low ambiguity, 1 = high ambiguity; 0 = United States, 1 = Japan; see Table 2 for the descriptive statistics and Table 3 for the correlations). Consistent with Study 2 and with Hypotheses 2 and 3, we found a Culture × Ambiguity interaction, *b* = −0.51, *t* = −3.96, *p* < 0.001, 95% CI [−0.763, −0.257], in addition to the main effect of Ambiguity, *b* = −0.20, *t* = −2.20, *p* = 0.028, 95% CI [−0.382, −0.022]. The main effect of Culture was not significant, *b* = 0.08, *t* = 0.80, *p* = 0.423, 95% CI [−0.121, 0.288]. In the more ambiguous situations, Japanese were less likely to help a stranger than Americans, *b* = −0.43, *t* = −5.62, *p* < 0.001, 95% CI [−0.576, −0.278]. However, in the low ambiguity situation, no cultural difference was found, *b* = 0.08, *t* = 0.08, *p* = 0.423, 95% CI [−0.121, 0.288].

**TABLE 2:** Means and standard deviations of main variables among American (*n* = 397) and Japanese participants (*n* = 399) in Study 3.

		United States			Japan		
	Ambiguity	Low (Tourist)	High (Tripping)	High (Luggage)	Low (Tourist)	High (Tripping)	High (Luggage)
1	Offering help	4.14 (0.90)	3.95 (0.96)	3.92 (0.92)	4.22 (0.73)	3.38 (0.94)	3.62 (0.73)
2	Perception of others’ need for help	4.42 (0.60)	3.57 (0.88)	3.78 (0.83)	4.05 (0.59)	2.71 (0.76)	3.32 (0.82)
3	Expectation of positive outcome for other	4.32 (0.62)	3.80 (0.64)	4.09 (0.56)	3.98 (0.61)	3.34 (0.55)	3.77 (0.50)
4	Expectation of positive outcome for self	3.73 (0.63)	3.59 (0.63)	3.72 (0.65)	3.49 (0.52)	3.12 (0.48)	3.29 (0.46)
5	Expectation of negative outcome for other	2.16 (1.00)	2.65 (0.80)	2.42 (0.84)	2.03 (0.58)	2.64 (0.55)	2.35 (0.61)
6	Expectation of negative outcome for self	2.02 (0.98)	2.37 (0.94)	2.30 (0.95)	2.03 (0.61)	2.48 (0.56)	2.23 (0.57)

*Values in parentheses are standard deviations.*

**TABLE 3:** Correlations of main variables among American (*n* = 397) and Japanese participants (*n* = 399) in Study 3.

		1	2	3	4	5	6
1	Offering help	–	0.561***	0.541***	0.482***	−0.512***	−0.545***
2	Perception of others’ need for help	0.264***	–	0.628***	0.458***	−0.530***	−0.433***
3	Expectation of positive outcome for other	0.188***	0.599***	–	0.681***	−0.700***	−0.627***
4	Expectation of positive outcome for self	–0.007	0.488***	0.528***	–	−0.518***	−0.549***
5	Expectation of negative outcome for other	−0.626***	−0.182***	−0.247***	0.064	–	0.798***
6	Expectation of negative outcome for self	−0.742***	−0.177***	−0.206***	0.073	0.859***	–

Values below the diagonals are for the United States and those above the diagonals are for Japan. ***p < 0.001.

Next, using PROCESS model 8 (Hayes, 2018), we examined whether the perception of other’s need for help and the expected outcomes for the target and the self explained the cultural difference in offering help, especially in the high ambiguous situations (Hypothesis 4; see Figure 3). Perception of other’s need for help was the only significant mediator in the model. The perceived need for help accounted for the cultural difference in offering help in both the high (*b* = −0.15, 95% CI [−0.21, −0.09]) and low ambiguity situations (*b* = −0.09, 95% CI [−0.14, −0.05]). As expected, the indirect effect was stronger in the high than low ambiguity situations, as indicated by a significant index of moderated mediation, *b* = −0.06, 95% CI [−0.12, −0.01]. The moderated mediation was due to Japanese respondents perceiving less need of help in the high (*b* = −0.62, 95% CI [−0.76, −0.49]) than low ambiguity situations (*b* = −0.38, 95% CI [−0.56, −0.19]) relative to American respondents.

Compared to American respondents, Japanese respondents were less likely to expect positive outcomes for others and the self in both high (*b*_*other*_ = −0.35, 95% CI [−0.45, −0.25]; *b*_*self*_ = −0.44, 95% CI [−0.53, −0.34]) and low ambiguity situations (*b*_*other*_ = −0.35, 95% CI [−0.49, −0.20]; *b*_*self*_ = −0.24, 95% CI [−0.38, −0.11]). However, the expectation of positive outcome for the self and for other did not predict offering help (*b*_*other*_ = −0.02, 95% CI [−0.13, −0.09]; *b*_*self*_ = 0.01, 95% CI [−0.09, 0.10]), resulting in non-significant indirect effects. Japanese and Americans did not differ in their expectations of negative outcomes for others and the self in either high (*b*_*other*_ = −0.07, 95% CI [−0.20, 0.06]; *b*_*self*_ = 0.01, 95% CI [−0.13, 0.14]) or low ambiguity situations (*b*_*other*_ = −0.13, 95% CI [−0.31, 0.05]; *b*_*self*_ = 0.01, 95% CI [−0.17, 0.20]). Although the expectation of negative outcome for the self was significantly associated with a lower tendency to offer help (*b* = −0.68, 95% CI [−0.78, −0.57]), neither indirect effect was significant.

The results of Study 3 provided further support to Hypotheses 2 and 3 that Japanese are less likely than Americans to offer help to a stranger but that the cultural difference emerges only in ambiguous situations. Consistent with Hypothesis 4, this cultural difference in helping was explained, at least partially, by Japanese lower tendency to perceive others as needing or wanting help. Surprisingly, Japanese lower tendency to expect positive outcomes for the target did not explain the cultural difference in offering help. We speculate that the high correlation between the perception of other’s need for help and expectation of positive outcomes for others accounts for this null finding (*r*s = 0.60 in the United States and.63 in Japan). When we reanalyzed the model in Figure 3 after excluding the perception of other’s need for help, the expectation of positive outcome for others significantly explained the cultural difference in offering help in both the high (*b* = −0.04, 95% CI [−0.08, −0.004]) and low ambiguity situations (*b* = −0.04, 95% CI [−0.08, −0.01]).

Another important contribution of Study 3 was in showing that perceiving fewer benefits or greater costs to the self did *not* explain the cultural difference in helping in the ambiguous situations. These findings are consistent with Study 1, which showed that concerns over the burden or risks for the self were not majors reasons for Japanese decisions to refrain from helping a stranger in ambiguous situations.

## Study 4: Is This “Helping” Helpful?

Studies 1–3 build on and extend earlier work on cultural variation in providing support and aid to known others. We find that in ambiguous situations, Japanese appear to be less likely than Americans to provide unsolicited support to a stranger because of their greater concern with the needs and wants of the stranger. If meeting others’ needs and expectations determines whether an intervention can be interpreted as helping and if strangers’ needs and expectations are unclear, are Japanese less likely than Americans to perceive their actions as helping and as having a positive impact? In Study 4, participants imagined taking actions to help strangers that resulted in either positive, negative, or neutral outcomes and rated how much the action was helpful and successful, and also how responsible they felt for the impact of the action. The normative ideas and practices of Japanese cultural contexts foster the construal that one’s actions are always in relation to others and encourage people to infer what others need and want from a given situation. With unknown others, this inference is extremely challenging, even if the impact of one’s actions and the likely outcome appears on the surface to be positive. Thus, we predicted that relative to Americans, Japanese respondents would feel more responsible for their actions to provide aid to a stranger and less likely to believe these actions were helpful and successful when the outcome of their actions is negative, but also when the outcome is positive (Hypothesis 5). We also hypothesized that people in the Japanese context would be less likely than those in the American context to say they would intervene again and that their lower tendencies to perceive their action as helpful and successful would explain this cultural difference in future intervention.

### Method

#### Participants

Our target number of participants for each sample was 200. *A priori* power analysis using G*Power 3 (Faul et al., 2009) with small effect size (0.20), alpha.05, and power at 95% recommended a total sample size of 390. For the United States sample, 203 participants were recruited through Amazon’s MTurk. All participants had the option to submit or withdraw their data at the end of the survey. One participant asked to have their data withdrawn, leaving a final sample of 202 (39.6% women). Participants’ age ranged from 20 to 71, with a mean of 34.33 (*SD* = 9.32). The majority of participants indicated their race as White or European American (73.8%), with 13.4% identifying as Black or African American, 5.0% as Asian, and 4.0% as Hispanic or Latino(a). Most participants (96.5%) indicated being born in the United States The six participants who were not born in the United States indicated they have lived in the United States between 4 and 37 years.

For the Japanese sample, 222 participants were recruited through Lancers. Sixteen left the majority of the survey blank and two wished to withdraw their data, resulting in a final sample of 204 (52.0% women). Participants’ ages ranged from 19 to 70, with a mean of 40.54 (*SD* = 9.85). All but three participants reported their nationality as being Japanese.

#### Scenarios

Participants read three scenarios that involved intervening on behalf of another person unknown to them (e.g., “A person trips over a curb and falls to the ground (…). You reach out to pull the person”). The scenarios either all ended with positive outcomes (e.g., “The person gets up and thanks you”), with negative outcomes (e.g., “the person seems embarrassed to be the center of attention”), or with no information about the outcome. The scenarios were created by the authors to reflect situations that are relatively common in and would instigate intervention in both United States and Japanese contexts (see Supplementary Material for full scenarios). The order of the three scenarios was randomized within participant.

#### Measures

The mean ratings of the three scenarios were used in the analyses. Participants rated from 1 (*not at all*) to 5 (*very much*), how much their behavior could be called a helping behavior (α_United States_ = 0.70, α_Japan_ = 0.67), the perceived outcome of their behavior (“How successful do you think your behavior was?” and “How positive/negative were the consequences of your behavior?” α_United States_ = 0.91, α_Japan_ = 0.83), how much they felt they had caused the outcome (“How much do you think you have caused the person’s current situation?” and “How responsible do you feel for what happened following your behavior?”) which was asked in the positive and negative outcome conditions (α_United States_ = 0.83, α_Japan_ = 0.64), and how likely they would do the same behavior if they were in that situation again (α_United States_ = 0.69, α_Japan_ = 0.76). The questionnaire was first developed in English, then translated into Japanese by a bilingual psychologist and back translated by a bilingual assistant. The two bilinguals and one native speaker of English discussed to solve any discrepancies.

### Results and Discussion

**FIGURE 4:**
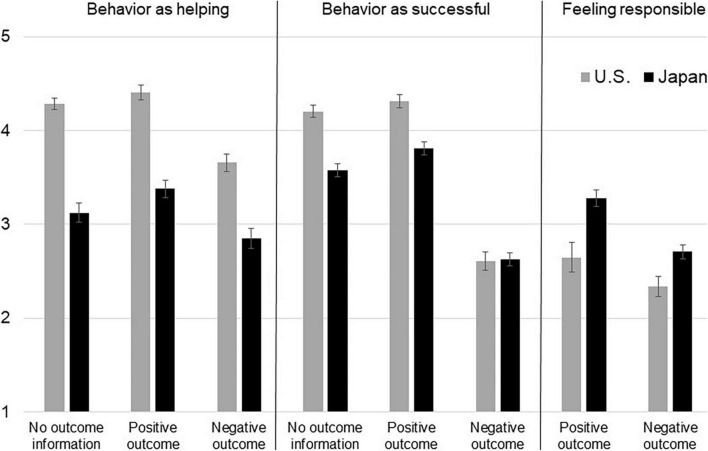
Japanese participants are less likely than Americans to describe their action as helping another, as successful, but more likely to feel responsible, regardless of the outcome (Study 4). Error bars represent standard errors.

Japanese associated their actions less with helping than Americans, *F*(2, 400) = 183.48, *p* < 0.001, η^2^ = 0.31 (see Figure 4 and Supplementary Material for the descriptive statistics). Moreover, consistent with Hypothesis 5, the effect of culture did not differ depending on the outcome of the behavior, *F*(2, 400) = 1.96, *p* = 0.143, such that Japanese were less likely than Americans to see their behavior as helping even when the outcome was described as positive, *t*(133) = 8.45, *p* < 0.001, *d* = 1.45. Japanese were also less likely than Americans to perceive their action as successful, *F*(2, 400) = 37.51, *p* < 0.001, η^2^ = 0.086. Although the effect of culture varied by outcome, *F*(2, 400) = 10.93, *p* < 0.001, η^2^ = 0.052, a *post hoc* comparison showed that Japanese perceived their actions as less successful than Americans even when the outcome was described as positive, *t*(133) = 4.931, *p* < 0.001, *d* = 0.85. Notably, Japanese also felt they had caused the outcome more so than Americans, *F*(1, 268) = 20.57, *p* < 0.001, η^2^ = 0.071, both when the outcome was described as positive, *t*(133) = −3.52, *p* = 0.001, *d* = 0.60 and negative, *t*(135) = −2.85, *p* = 0.005, *d* = 0.50.

Japanese were less likely than Americans to indicate that they would engage in the same action if they were in the same situation again, *F*(1, 400) = 139.78, *p* < 0.001, η^2^ = 0.259. The effect of culture did not differ by the outcome, *F*(1, 400) = 2.43, *p* = 0.089; Japanese were less eager than Americans to engage in a similar action again, even after they learned the outcome was positive, *t*(133) = 7.15, *p* < 0.001, *d* = 1.20. When we ran a mediation analysis using PROCESS (Hayes, 2018; model 4) with culture (0 = United States and 1 = Japan) as the predictor, perceived helpfulness, success, and responsibility as the mediators, and the motivation to intervene again as the outcome variable, Japanese lower tendencies to perceive their action as helpful and successful explained their lower likelihood of engaging in a similar action in the future (the indirect effects were −0.39, 95% CI [−0.54, −0.25] and −0.06, 95% CI [−0.14, −0.002], respectively). Perceiving themselves to have caused the outcome was not a mediator (indirect effect = −0.004, 95% CI [−0.0, 0.04]). In sum, Japanese rated their actions as less helpful and successful than American respondents and felt more responsible for their actions, and this was so even when the outcome was positive. In Japan, providing aid to another is less readily interpreted as helpful; this explains, at least partially, Japanese lower tendency to intervene in lives of strangers.

## General Discussion

Will this help be helpful? In four studies, we demonstrated that Japanese are less likely than Americans to offer unsolicited help to a stranger because of their greater concern with attunement to others’ wants and needs for help. Study 1 showed that Japanese were more likely than Americans to spontaneously mention others’ wants and needs for help as a reason for not offering help to strangers. Study 2 showed that the ambiguity about the need for help had a greater impact on the decision to offer help among Japanese than Americans. Study 3 showed that the Japanese lower tendency to perceive others as needing or wanting help and their lower certainty that their intervention would benefit others explained their lower tendency to offer help in ambiguous situations. Finally, Study 4 suggests that in Japan, any intervention on behalf of a stranger may be less readily construed as helping than in the United States contexts. Together, these results provide converging evidence that Japanese contexts encourage attention to others’ needs and wants when deciding if, when, and how to intervene on their behalf—the imperative captured succinctly by the Platinum Rule—treat others the way *they* want to be treated. Yet unless the situation and others’ needs and wants are clear, widespread adherence to this norm makes it less likely that Japanese will perform the type of prosocial actions that are labeled “helping” in North America.

Why do Japanese refrain from intervening when others’ needs are unclear? Japanese may not intervene on behalf of others because doing so can be unhelpful or even harmful if rooted only in one’s good intentions and do not consider the circumstances of the other and the impact of intervention. Giving inadequate or unwanted aid carries the potential to harm, embarrass, or harm the person’s face (Kim et al., 2008; Miller et al., 2017). Moreover, receiving help can create a debt, and Japanese may worry about repaying this debt more than Americans (Miller et al., 2017). Thus, in Japan where transgressions and their impact are weighted more heavily than the intention behind an action (Feinberg et al., 2018), intervening with a stranger may not be as closely associated with the prosocial concept of “helping” as it is for Americans. Moreover, in Japanese cultural contexts there is often an acknowledgment and elaboration of the idea that in some circumstances, a non-intervention can be more helpful than an intervention (Mitsuno and Miura, 2010; Yamamura, 2013). What appears as “doing nothing” from the perspective of an independent model of agency may, in fact, be an effortful, active, agentic response to a situation when perceived through an interdependent model of agency in which one’s actions are always in relation to others.

One could argue that Japanese do not intervene because they are more apathetic toward strangers or too risk-averse to intervene and that they may be simply justifying their inaction by claiming that others do not want or need their help. Although this explanation is also plausible, we believe that it does not constitute the main reason for the non-intervention. In Study 1, we found no cultural differences in individual ratings of uncertainty avoidance and only a small percentage of Japanese (<10%) referred to future burdens or potential risks to the self as explanations for non-intervention. Moreover, Study 3 showed that self-interest did not explain the cultural difference in helping in ambiguous situations. In Study 4, Japanese respondents reported feeling more responsible for the consequences of their actions toward strangers than did Americans, suggesting they are not more apathetic to strangers. Similarly, ingroup favoritism or cultural embeddedness cannot fully explain these differences. Though situations with strangers are often inherently more ambiguous than those with close others, only a small percentage of respondents (11.5%) listed the person being a stranger as an explanation for non-intervention (Study 1). Furthermore, when the need for help was clear, Japanese helped as much as or sometimes more than Americans (Studies 2 and 3). Though people in Japanese contexts may avoid asking for help or burdening others (Kim et al., 2008), this work suggests that if help is needed, people can ask and expect to get it.

This work contributes to a growing understanding that there are multiple forms of agency (e.g., Markus and Kitayama, 2003; Kitayama et al., 2006; Stephens et al., 2009; Markus, 2016). With the independent model of agency, the focus of questions about if, when, and how to act is relatively concentrated on the self—one’s intention to help, the belief that one wants to or can help. The individual who performs the action determines others’ needs for help by projecting their own needs as well as the meaning of their intentions and actions. Accordingly, when an individual intervenes with the intention to help others, that action can be readily interpreted as helping, even if the recipient resents or resists the intervention. With a more interdependent model of agency, the focus is relatively holistic, going beyond the actor’s intention and capacity to encompass the relation between the actor’s action and its potential impact on the recipient in the particular situation. An action is considered helpful only to the extent that it meets others’ needs for help. People in Japanese contexts tend to perceive unwanted or poorly executed “help” to be less helpful than Americans because their action takes meaning only within the situational and relational context within which it is embedded. In fact, in an interdependent cultural context, “doing nothing” may be perceived as helping when it is considered the most beneficial behavior for others (Mitsuno and Miura, 2010; Yamamura, 2013).

These findings shed some more light on the still understudied ways of being that are common outside of individualist, middle class, Western contexts (Adams et al., 2019). While the relatively greater attention paid to the situation and to others in some cultural contexts outside the West have been well-documented (e.g., for reviews see Cohen and Kitayama, 2018), the consequences of this very general behavioral orientations for specific actions, and particularly for actions that appear universal—as in the case of providing aid to others in contexts—are not examined and well understood. Many models of agency and the normative ideas and practices they reflect about if, when, how and why to act have yet to be described and examined for their psychological and behavioral correlates and consequences (Markus and Hamedani, 2018). Solidifying these findings would benefit from more controlled experimental work, as well as larger more representative samples.

Our findings are limited in that we only examined a subset of situations where people must decide whether or not to offer a helping hand to a stranger. We only examined offering help to a stranger because an encounter with a stranger is inherently more ambiguous than an interaction with a close other; whether situational ambiguity reduces helping even among close others remains an open question. The scenarios in Study 3 varied in ambiguity as well as in the type of setting and behavior required for helping; it would be important to replicate the findings using scenarios involving other settings or scenarios that only vary in the ambiguity. Finally, our research only examined self-reported intention to offer help. We hope that a follow-up study clarifies whether people in the United States and Japan differ in how often they actually help a stranger and how they offer help (i.e., Do people simply ask if a person needs help? Do people actually engage in an action to help the person?).

Future research might also productively examine how Japanese determine others’ needs for help. There are instances where a person requests help that is not needed or that could be harmful (e.g., a student who asks a classmate to copy homework), as well as instances where a person does not request help even when she unambiguously appears to need it (e.g., a suicidal person who asks to be left alone). How Japanese offer or refrain help in these morally fraught situations will elucidate the complexity of helping in an interdependent cultural context. Overall, our research demonstrates how seemingly simple actions such as providing aid to another who appears to require it, are often the result of complex culturally afforded processes of meaning-making. Actions that reflect models of agency different from one’s own can appear selfish, deficient, irrational, and immoral.

This research also highlights an American penchant for “doing something,” even if the impact of the action or the needs of others is not clear, and/or when the help is unsolicited. This behavioral orientation has multiple significant implications, in particular, for foreign policy and international relations. As inequality, climate change, and pandemics increasingly highlight global interdependence and demand international cooperation, a more nuanced understanding of how and why people act is essential for avoiding conflict and for fostering intercultural communication and coordination (Thomas et al., 2020).

## Data Availability Statement

The original contributions presented in the study are publicly available. This data can be found here: https://data.mendeley.com/datasets/sfv46y3wmm/1.

## Ethics Statement

The studies involving human participants were reviewed and approved by Hosei University Ethics Advisory Committee and Stanford IRB. The patients/participants provided their written informed consent to participate in this study.

## Author Contributions

All authors developed the study concept, contributed to the study designs, drafted the manuscript, provided the critical revisions, and approved the final version of the manuscript for submission. YN and CH performed the testing, data collection, and data analysis.

## Conflict of Interest

The authors declare that the research was conducted in the absence of any commercial or financial relationships that could be construed as a potential conflict of interest.

## Publisher’s Note

All claims expressed in this article are solely those of the authors and do not necessarily represent those of their affiliated organizations, or those of the publisher, the editors and the reviewers. Any product that may be evaluated in this article, or claim that may be made by its manufacturer, is not guaranteed or endorsed by the publisher.

## Footnotes

1 These two “rules” are widely known across cultural contexts and commonly quoted and invoked in business, educational, counseling, and religious contexts. They are also common in various forms of diversity training that emphasize trying to take the perspective of the other. The sources of these rules are murky and they are multiply attributed. Some authors locate both rules in the Bible, others claim the Bible only supports the Golden rule. Recent relevant references for further explanation include Wattles (1996), Bennett (1998), and Christensen and Medina (2016).
