# Supplementary material for: Will This Help Be Helpful? Giving Aid to Strangers in the United States and Japan
Source: Front Psychol. 2022 Jan 25;12:784858. doi: 10.3389/fpsyg.2021.784858 (PMC8821533; doi:10.3389/fpsyg.2021.784858)
Supplement: Supplementary file 1 [file Table_1.DOCX]

***Supplementary Materials***

1. **Measures used in Study 3**

Offering help:

- How likely would you be to help the person up from the ground in this situation? (1 = *very unlikely*, 5 = *very likely*)
- How hesitant would you be to help the person [up] in this situation? (1 = *not at all hesitant*, 5 = *extremely hesitant*; reversed)
- How stressful would it be to make the decision to help or not in this situation? (1 = *not stressful at all*, 5= *very stressful*; reversed).

Perception of other’s need for help:

- How much do you think the person needs help [up/put their bag in the overhead bin/to give up your seat] in this situation? (1 = *definitely not*, 5 = *definitely yes*)
- If you were the person [who tripped/struggling with your bag/who looked pregnant], how much would you want to be helped? (1 = *definitely not*, 5 = *definitely yes*)

Expectation of positive outcome for the target:

- How likely is it that there would be positive outcomes to the person if you were to help in this situation? (1 = *extremely unlikely*, 5 = *extremely likely*)
- If you were to help the person up, how possible is it that the person would feel...
  - grateful, happy, taken care of, relieved. (1 = *extremely unlikely*, 5 = *extremely likely*)

Expectation of positive outcome for the self:

- How likely is it that there would be positive outcomes to you if you were to help in this situation? (1 = *extremely unlikely*, 5 = *extremely likely*)
- If you were to help, how possible is it that you would feel...
  - powerful, in control, appreciated, proud, good, important, admired, competent (1 = *extremely unlikely*, 5 = *extremely likely*)
- If you were to help, how possible is it that your help would be perceived as...
  - helpful, welcomed, supportive, caring (1 = *extremely unlikely*, 5 = *extremely likely*)

Expectation of negative outcome for the target:

- How likely is it that there would be negative outcomes to the person if you were to help in this situation? (1 = *extremely unlikely*, 5 = *extremely likely*)
- If you were to help the person up, how possible is it that the person would feel...
  - embarrassed, offended, inferior, uncomfortable, angry, helpless, the focus of unwanted attention (1 = *extremely unlikely*, 5 = *extremely likely*)

Expectation of negative outcome for the self:

- How likely is it that there would be negative outcomes to you if you were to help in this situation? (1 = *extremely unlikely*, 5 = *extremely likely*)
- If you were to help, how possible is it that you would feel...
  - uncomfortable, disrespectful, presumptuous, guilty, incompetent, regretful, embarrassed, self-promoting (1 = *extremely unlikely*, 5 = *extremely likely*)
- If you were to help, how possible is it that your help would be perceived as...
  - burdensome, awkward, harmful, unhelpful, self-promoting (1 = *extremely unlikely*, 5 = *extremely likely*)

1. **Full scenarios used in Study 4**
2. Imagine you are walking down a semi-crowded street. Ahead of you, a person trips over a curb and falls to the ground. You are worried that the person is injured. You reach out to pull the person up.

- Positive outcome: The person gets up and thanks you.
- Negative outcome: … but the person seems embarrassed to be the center of attention.

1. Imagine you are on an airplane. Ahead of you is a person who is struggling to put her luggage in the overhead bin. The person seems frustrated. You reach out to push the bag into the bin.

- Positive outcome: The bag goes in and the person thanks you.
- Negative outcome:… but the bag seems too large to fit in the bin. The cabin attendant says the bag needs to be checked.

1. As you are walking down the street, a tourist seems to be looking for directions to the airport. They seem to be in a hurry. You give the person directions to a shortcut.

- Positive outcome: The person thanks you and successfully reaches their destination on time.
- Negative outcome: Later on you find out there was a traffic jam on that particular shortcut you suggested and realize that the person likely missed their flight.

1. **Means and Standard Deviations of Variables among Americans (*n* = 202) and Japanese (*n* = 204) in Study 4**

|  | US | | | | Japan | | | |
| --- | --- | --- | --- | --- | --- | --- | --- | --- |
|  | No information (*n* = 67) | Positive outcome  (*n* = 67) | Negative outcome (*n* = 68) | Total (*n* = 202) | No information (*n* = 67) | Positive outcome (*n* = 68) | Negative outcome (*n* = 69) | Total (*n* = 204) |
| Behavior as helping | 4.28 | 4.41 | 3.66 | 4.11 | 3.12 | 3.38 | 2.85 | 3.12 |
|  | (0.50) | (0.64) | (0.76) | (0.72) | (0.83) | (0.76) | (0.89) | (0.85) |
| Behavior as successful | 4.10 | 4.23 | 2.38 | 3.56 | 3.20 | 3.47 | 2.22 | 2.96 |
|  | (0.61) | (0.72) | (0.97) | (1.15) | (0.70) | (0.79) | (0.62) | (0.88) |
| Desire to help again | 4.26 | 4.46 | 3.67 | 4.13 | 3.16 | 3.56 | 2.98 | 3.24 |
|  | (0.65) | (0.62) | (0.81) | (0.77) | (0.85) | (0.82) | (0.80) | (0.85) |
| Feeling responsible | *--* | 2.65 | 2.33 | 2.49 ^a^ | *--* | 3.28 | 2.88 | 3.08 ^b^ |
|  | -- | (1.30) | (0.90) | (1.12) | -- | (0.72) | (0.70) | (0.74) |

Note. Values in parentheses are standard deviations. ^a^ mean based on *n* = 135; ^b^ mean based on *n* = 137
